# Determining the aortic root diameter necessitating replacement during acute type A aortic dissection repair

**DOI:** 10.1016/j.xjon.2026.101804

**Published:** 2026-04-08

**Authors:** Akihiro Yoshitake, Naohiko Oki, Osamu Kinoshita, Takayuki Gyoten, Yuta Kanazawa, Yuto Hori, Hiroaki Aizawa, Taro Kuroda, Yu Kumagai, Toshihisa Asakura

**Affiliations:** Department of Cardiovascular Surgery, Saitama Medical University International Medical Center, Saitama, Japan

**Keywords:** acute type A aortic dissection, aortic root diameter, root preservation

## Abstract

**Objectives:**

To evaluate long-term outcomes after root preservation and identify the root diameter warranting concomitant replacement during acute type A aortic dissection (ATAAD) repair.

**Methods:**

Between January 2010 and December 2023, 681 patients with ATAAD underwent surgical repair. After excluding bicuspid aortic valve and connective tissue disorders, 42 patients underwent aortic root replacement or repair and 594 patients underwent ascending or total arch replacement with preservation of the native root. The patients were divided into 3 groups according to the aortic root diameter: <45 mm (group A; 535 patients), ≥45 and <50 mm (group B; 41 patients), and ≥50 mm (group C; 18 patients).

**Results:**

No significant differences in perioperative outcomes, specifically in-hospital mortality and stroke, were observed among the 3 study groups. The actual survival rates at 5 years and 10 years were 76.6% and 66.4% in group A, 79.2% and 66.0% in group B, and 94.1% and 44.1% in group C (log-rank*P* = .86). The rates of freedom from proximal aortic reoperation at 5 years and 10 years were 98.0% and 93.2% in group A, 92.3% and 92.3% in group B, and 72.0% and 28.0% in group C (log-rank *P* < .0001).

**Conclusions:**

During surgery for ATAAD, an aortic root with a diameter ≥50 mm should be replaced, given significantly higher 5- and 10-year proximal aortic reoperation rates. In patients with aortic root diameters <50 mm, even if ≥45 mm, the aortic root may be preserved.

**Figure:**
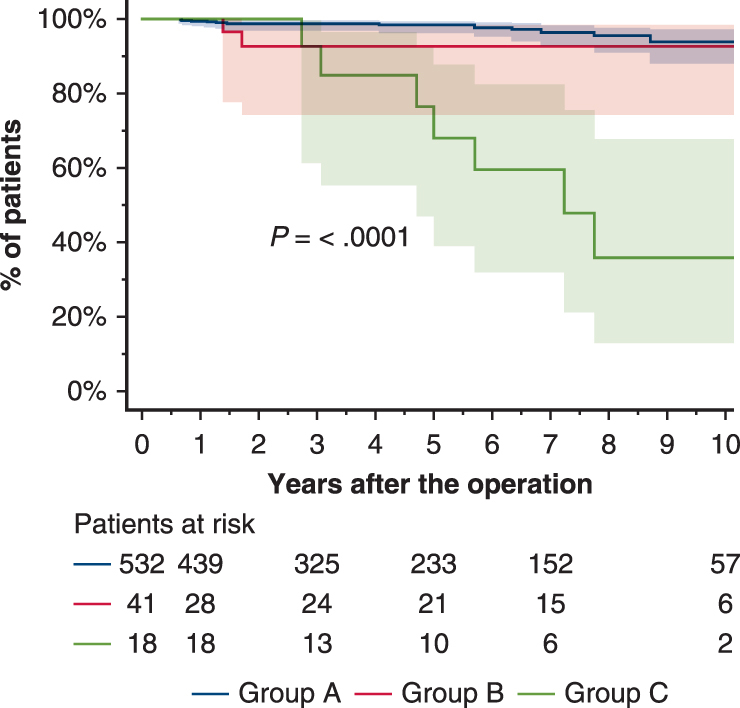
Freedom from proximal reoperation after operation for acute type A aortic dissection.

**Central Message**

During acute type A aortic dissection surgery, roots <50 mm may be preserved, including those 45 to 49 mm.

**Perspective**

A 50-mm cutoff may help guide selective root replacement during ATAAD repair while avoiding unnecessary operative complexity.

Acute type A aortic dissection (ATAAD) is a cardiovascular emergency that requires immediate surgical intervention. The primary goal of surgery is to resect the primary entry tear and prevent rupture or malperfusion while minimizing the operative risk. Recent debates have addressed when to perform hemi-arch replacement or total arch replacement,^1^^,^^2^ and the usefulness of the frozen elephant trunk technique has been widely reported.^3^^,^^4^ Nonetheless, the optimal management of the aortic root during ATAAD repair remains controversial.

Current surgical guidelines^5^^,^^6^ recommend elective aortic root replacement when the diameter reaches 50 to 55 mm, a threshold derived largely from data on chronic aneurysmal disease rather than on acute dissection. An aortic root diameter ≥45 mm may confer a higher risk of late reintervention^7^; however, this observation is based on limited evidence, not reaching a level that warrants a definitive recommendation. Several imaging studies, including the International Registry of Acute Aortic Dissection,^8^ have shown that many dissections occur at diameters below these thresholds. These findings suggest that aortic wall fragility or medial degeneration, rather than aortic wall size alone, may predispose patients to acute dissection. Furthermore, once the dissection extends into the aortic root, late complications, such as progressive dilation, recurrent aortic regurgitation, and pseudoaneurysm formation, may occur if the root is preserved.^9^^,^^10^

Conversely, root replacement during an acute operation increases the technical complexity, cardiopulmonary bypass and myocardial ischemic times, and perioperative risk.^11^^,^^12^ Therefore, surgeons often face the dilemma of replacing a dissected but moderately dilated root to prevent late complications or preserving it to reduce operative mortality. Although several studies have compared root replacement and preservation strategies,^13^, ^14^, ^15^ few have specifically examined the prognostic significance of moderate root dilatation in ATAAD.^7^

Given this uncertainty, determining the appropriate threshold for aortic root replacement during ATAAD repair remains a critical issue. Therefore, in the present study, we aimed to evaluate the long-term outcomes of patients undergoing aortic root preservation and to identify the aortic root diameter at which concomitant root replacement should be considered during surgery for ATAAD.

## Methods

### Study Population

In this study, ATAAD surgery was defined as surgical repair performed within 14 days of symptom onset. Between January 2010 and December 2023, 681 consecutive patients underwent surgical repair for ATAAD at our institution. We excluded patients with bicuspid aortic valve or connective tissue disorders, such as Marfan syndrome or Loeys-Dietz syndrome. These patients were excluded because their underlying aortic pathology and surgical thresholds differ substantially from those of patients with typical nonsyndromic ATAAD. Ultimately, 636 patients had available preoperative computed tomography (CT) data for precise aortic root measurement and were included in the study.

Among the 636 patients, 42 (6.6%) underwent aortic root replacement or repair based on the intraoperative findings of extensive root enlargement, intimal tears involving the sinus of Valsalva, or moderate enlargement with severe aortic regurgitation. In this study, we focused on the remaining 594 patients, who underwent ascending or total arch replacement with preservation of the native root, to evaluate the impact of aortic root diameter on long-term outcomes.

### Measurement of Aortic Root Diameter

Preoperative contrast-enhanced CT images were reviewed in the routinely reconstructed axial, sagittal, and coronal planes. The aortic root diameter was measured on the plane in which the left ventricular outflow tract, aortic valve, sinus of Valsalva, and proximal ascending aorta were visualized simultaneously. The maximal diameter within the selected plane was recorded. The measurements were performed by experienced cardiovascular surgeons at our institution according to a consistent review protocol. All immediate postoperative CT scans were examined at our institution using the same measurement protocol. A small proportion of patients underwent long-term follow-up imaging at outside institutions; in such cases, the reported measurements were used, which may represent a potential source of variability. Patients were classified into 3 groups according to aortic root diameter: group A, <45 mm; group B, ≥45 mm and <50 mm; and group C, ≥50 mm.

### Surgical Strategy

The main surgical procedures have been described in our previous publications.^3^ Surgical decision making was based not only on the aortic root diameter but also on the severity of aortic regurgitation as assessed by preoperative echocardiography. Aortic root replacement was performed in patients with the following presentations^1^: aortic root ectasia or markedly dilated aortic roots (usually ≥5 cm); (2) primary intimal tear involving the sinuses of Valsalva; or (3) moderate enlargement with severe aortic regurgitation. Patients with severe aortic regurgitation and a normal root diameter underwent AVR alone.

For the dissected aortic root, the primary entry tear was resected, BioGlue was applied, and felt strips were placed on the inner and outer layers to reinforce the dissected wall before performing the anastomosis with the graft. A proximal anastomosis was created approximately 1.5 cm distal to the sinotubular junction in most cases.

### Follow-up and Endpoints

Enhanced CT was performed 1 week after surgery. After discharge, follow-up CT was performed annually. Data were recorded prospectively, and the patients were followed-up in the outpatient unit of our institution. Telephone interviews were conducted in patients who underwent follow-up at another hospital. The mean duration of follow-up was 5.3 years.

The primary endpoint of the study was freedom from proximal aortic reoperation. The secondary endpoints included overall survival and aortic-related death. Reoperation was defined as any procedure involving the aortic root or ascending aorta after the index surgery.

### Statistical Analysis

Continuous variables are expressed as the mean ± standard deviation and were compared using one-way analysis of variance or the Kruskal-Wallis test, as appropriate. Categorical variables were analyzed using the χ^2^ test or Fisher exact test.

Survival, freedom from aortic-related death, and freedom from proximal reoperation were estimated using the Kaplan-Meier method, and the log-rank test was used to perform intergroup comparisons. The independent predictors of proximal aortic reoperation were identified using multivariate Cox proportional hazards regression analysis, incorporating clinically relevant covariates such as age, sex, preoperative aortic regurgitation, and root diameter. Statistical significance was set at *P*< .05. Statistical analyses were performed using JMP Student Edition 19 (SAS Institute).

### Ethical Considerations

The Institutional Review Board of Saitama Medical University International Medical Center approved this study (approval 2024-081, approved October 2, 2024). The requirement for informed written consent was waived owing to the study’s retrospective observational design. All patient data were anonymized before analysis.

## Results

### Patient Characteristics

Table 1 summarizes the baseline characteristics of the study cohort. The 594 patients who underwent aortic root preservation had a mean age of 66.9 ± 12.7 years, and 39.6% were female. Of these 594 patients, 535 were classified according to aortic root diameter as group A (<45 mm), 41 as group B (≥45 mm and <50 mm), and 18 as group C (≥50 mm).

**Table 1:** Patient characteristics

Variables	Group A (N = 535)	Group B (N = 41)	Group C (N = 18)	*P* value
Age, y, mean ± SD	67.2 ± 12.7	64.0 ± 13.5	61.3 ± 11.8	.053
Male sex, n (%)	265 (49.5)	37 (90.2)	15 (83.3)	<.0001
CKD requiring hemodialysis, n (%)	17 (3.2)	1 (2.4)	0 (0)	.544
Coma/hemiparesis, n (%)	43 (8.1)	3 (7.3)	1 (5.6)	.906
Preoperative intubation, n (%)	41 (7.7)	1 (2.4)	2 (11.1)	.309
Cardiac tamponade, n (%)	82 (15.7)	6 (14.6)	3 (16.7)	.978
Malperfusion, n (%)	178 (33.6)	17 (41.5)	10 (55.6)	.102
AR (>mild), n (%)	94 (19.0)	10 (26.3)	7 (43.8)	.033
DeBakey 1, n (%)	347 (64.9)	29 (70.7)	15 (83.3)	.211
DeBakey 2, n (%)	78 (14.6)	3 (7.3)	3 (16.7)	.417
DeBakey 3 retro, n (%)	110 (20.6)	9 (22.0)	0 (0)	.096
Patent false lumen, n (%)	352 (65.4)	28 (66.7)	17 (94.4)	.037
Dissection involving aortic root, n (%)	415 (77.6)	40 (97.6)	17 (94.4)	.003
ULP, n (%)	38 (7.1)	4 (9.5)	0 (0)	.218
Thrombosed false lumen, n (%)	140 (26.5)	9 (22.0)	1 (5.6)	.117

*CKD*, Chronic kidney disease; *AR*, aortic regurgitation; *ULP*, ulcer-like projection.

Significant differences were observed among the 3 groups in terms of age (67.2 ± 12.7 years in group A, 64.0 ± 13.5 years in group B, 61.3 ± 11.8 years in group C; *P* = .0164) and sex distribution (*P* < .0001). The incidence of moderate-to-severe preoperative aortic regurgitation also differed significantly across the 3 groups (*P* = .033), being the highest in group C. The rate of dissection involving the aortic root also differed significantly among the groups (*P* = .003).

### Operative Findings

The operative data are summarized in Table 2. The extent of distal repair (hemiarch vs total arch replacement) and proportion of concomitant procedures were similar among the study groups. Concomitant CABG was performed in 22 patients, in 11 cases due to preoperative coronary malperfusion associated with the dissection. The remaining procedures were performed based on intraoperative assessment of myocardial ischemia or difficulty in weaning from cardiopulmonary bypass. No CABG was required due to coronary button–related technical complications.

**Table 2:** Procedural details

Procedure	Group A (N = 535), n (%)	Group B (N = 41), n (%)	Group C (N = 18), n (%)	*P* value
Hemi arch replacement	254 (49.0)	19 (48.7)	8 (50.0)	.996
Partial arch replacement	31 (6.0)	3 (7.7)	1 (6.3)	.912
Total arch replacement	233 (45.0)	17 (43.6)	7 (38.9)	.942
With FET	217 (40.9)	16 (39.0)	7 (38.9)	.962
Concomitant procedure	58 (10.9)	6 (14.6)	1 (5.6)	.576
CABG	21 (4.0)	1 (2.4)	0 (0)	.438
Aortic valve replacement	10 (1.9)	1 (2.4)	0 (0)	.689

*FET*, Frozen elephant trunk; *CABG*, coronary artery bypass grafting.

### Early Outcomes

The overall in-hospital mortality rate of the 594 patients was 4.7% (28 deaths). No significant differences were observed among the groups (4.5% [n = 24] in group A, 9.8% [n = 4] in group B, and 0% in group C; *P* = .199). The incidence of postoperative stroke was 6.6% overall (39 cases) and was comparable across the 3 groups (6.5% in A, 4.9% in B, and 16.7% in C; *P* = .208) (Table 3).

**Table 3:** Surgical outcomes

Variable	Group A (N = 535)	Group B (N = 41)	Group C (N = 18)	*P* value
30-day death, n (%)	11 (2.1)	2 (4.9)	0 (0)	.395
In-hospital death, n (%)	24 (4.5)	4 (9.8)	0 (0)	.199
Neurologic dysfunction, n (%)	34 (6.5)	2 (4.9)	3 (16.7)	.208
Spinal cord ischemia, n (%)	2 (0.4)	0 (0)	0 (0)	.809
Renal failure on HD, n (%)	29 (5.5)	3 (7.3)	3 (16.7)	.133
Prolonged intubation (≥72 h), n (%)	252 (46.7)	26 (65.0)	9 (50.0)	.431
ICU stay, d, mean ± SD	12.1 ± 13.5	11.1 ± 7.0	12.8 ± 12.5	.864

*HD*, Hemodialysis; *ICU*, intensive care unit.

Residual dissection involving the aortic root was observed on immediate postoperative CT in 3 patients (0.51%). The diameter of the sinus of Valsalva decreased from 36.6 ± 4.4 mm to 35.3 ± 3.9 mm in group A (*P* < .0001), from 47.0 ± 1.2 mm to 43.1 ± 3.0 mm in group B (*P* = .881), and from 51.8 ± 1.6 mm to 46.8 ± 3.2 mm in group C (*P* = .586) (Table 4).

**Table 4:** Sinus of Valsalva diameter

Parameter	Group A	Group B	Group C	*P* value
Preoperative SOV	36.6 ± 4.4	47.0 ± 1.2	51.8 ± 1.6	<.0001
Postoperative SOV	35.3 ± 3.9	43.1 ± 3.0	46.8 ± 3.2	<.0001
*P* value (pre vs post)	<.0001	.881	.586	
Dissection residual	2 (0.4)	0 (0)	1 (6.3)	.152

*SOV*, Sinus of Valsalva.

### Late Outcomes

**Figure 1:**
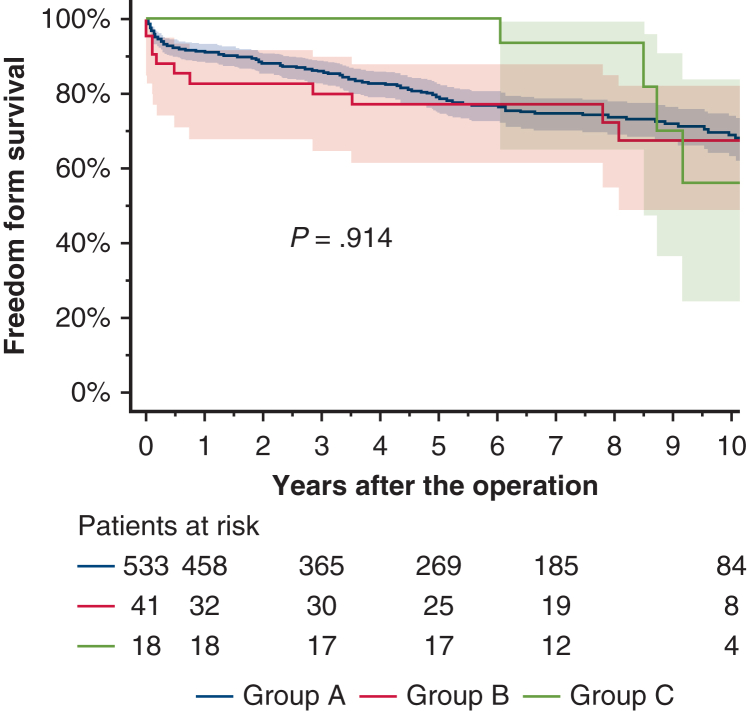
Survival curves. Shaded areas represent 95% confidence intervals.

The Kaplan-Meier estimated survival rates at 5 years and 10 years were 78.5% and 68.7% in group A, 77.0% and 67.3% in group B, and 100.0% and 56.0% in group C (log-rank *P* = .914) (Figure 1). The rates of freedom from aortic-related death at 5 years and 10 years were 93.4% and 91.0% in group A, 90.2% and 90.2% in group B, and 100% and 93.3% in group C (log-rank *P* = .867). No significant differences in overall or aortic-related survival were observed across the 3 groups.

### Proximal Aortic Reoperation

Proximal aortic events were defined as any reintervention involving the proximal aorta, including progressive root dilation, pseudoaneurysm formation at the proximal anastomosis, and redissection. During follow-up, proximal aortic reoperation was required in 22 patients. In group A, 11 reoperations (2.0%) were performed, including 10 for pseudoaneurysm formation at the sinotubular junction and 1 for redissection. In group B, 2 patients (4.8%) underwent reoperation, 1 with progressive root dilation and 1 with a pseudoaneurysm. In group C, 9 patients (45.0%) required reoperation owing to progressive root enlargement.

**Figure 2:**
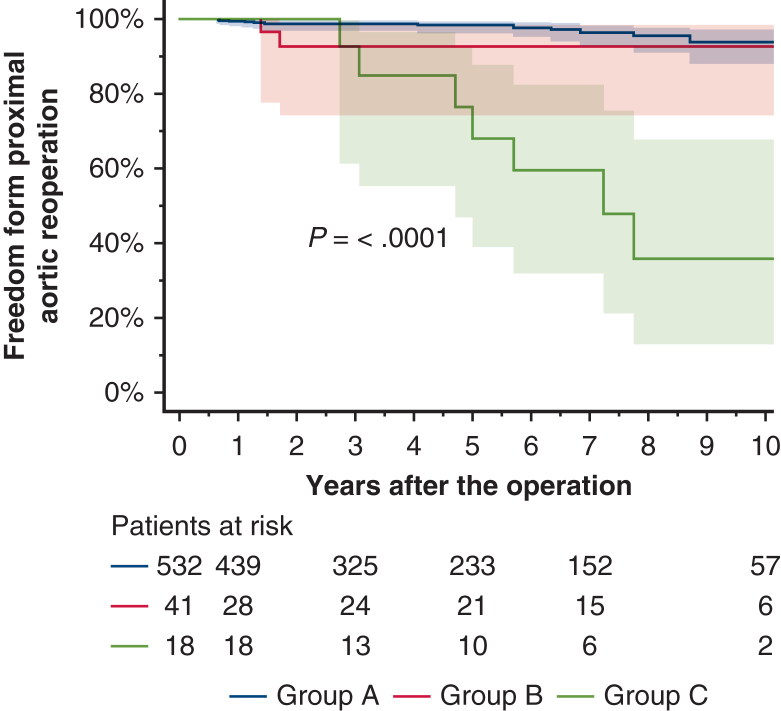
Freedom from proximal reoperation. Shaded areas represent 95% confidence intervals.

The rates of freedom from proximal reoperation at 5 years and 10 years were 98.3% and 94.6% in group A, 92.3% and 92.3% in group B, and 69.2% and 36.4%, in group C (Figure 2). The differences among the groups were highly significant (log-rank *P* < .0001).

In the multivariable Cox proportional hazards model, a preoperative aortic root diameter ≥50 mm was independently associated with an increased risk of long-term proximal aortic events compared with a diameter <45 mm (adjusted hazard ratio [aHR], 15.7; 95% confidence interval [CI], 4.9-50.6; *P* < .0001). In contrast, a root diameter of 45 to 49 mm was not independently associated with long-term aortic events compared to root diameters <45 mm (aHR, 2.90; 95% CI, 0.59-14.3; *P* = .19).

When analyzed as a continuous variable, preoperative aortic root diameter was significantly associated with long-term proximal aortic events (HR per 1-mm increase, 1.18; 95% CI, 1.10-1.27; *P* < .0001).

## Discussion

The results of this single-center study demonstrated that preservation of the native aortic root was generally safe and durable in patients undergoing repair for ATAAD when the acute diameter was <50 mm. Moderate dilatation (45-49 mm) was not associated with a higher risk of proximal reoperation or aortic-related mortality In contrast, roots ≥50 mm showed a marked increase in late root enlargement and reoperation. Continuous modeling also demonstrated a graded association between root diameter and proximal events, supporting the clinical relevance of the 50-mm threshold. Both progressive root dilation and proximal pseudoaneurysm required proximal reintervention and thus were analyzed together as proximal aortic events. No root rupture occurred during follow-up; however, reinterventions were performed for clinically significant proximal pathology, such as progressive dilation, pseudoaneurysm, redissection, or worsening aortic regurgitation. Therefore, the absence of rupture alone should not be interpreted as evidence that reoperation was unnecessary.

Previous reports have yielded inconsistent conclusions regarding the optimal management of the aortic root in ATAAD. Rylski and colleagues^16^ demonstrated that residual dissection or root ectasia predisposes to late dilation, whereas Vendramin and colleagues^7^ showed that conservative treatment can be safe when the diameter is ≤45 mm. Our present results extend these observations by demonstrating that even when the root diameter is moderately enlarged (45-49 mm), root preservation can achieve acceptable long-term durability, provided that structural disruption is not extensive.

The sharp increase in reoperation risk above 50 mm likely reflects the cumulative effects of medial degeneration, wall stress, and persistent false-lumen pressurization. When the dissected sinus wall is thin or partially destroyed, postoperative remodeling may exacerbate dilatation, particularly in roots that were already ≥50 mm.

Another observation was that the aortic root often appeared enlarged during the acute phase of dissection. In our series, postoperative measurements in the 45 to 49 mm group frequently regressed to <45 mm. This finding is consistent with previous imaging studies^17^ suggesting that root enlargement during acute dissection may reflect transient hemodynamic expansion due to false lumen pressurization rather than irreversible structural dilation. Therefore, moderate enlargement in the acute setting should not automatically mandate root replacement in the absence of significant structural destruction.

International registries^18^^,^^19^ suggest that aortic root replacement is performed more frequently in Western countries than in Japan.^20^ Differences in baseline aortic size, institutional philosophy, and surgical experience may contribute to this variation.^21^ Although indexing by body surface area may refine risk assessment, absolute diameter remains the most practical parameter during emergency ATAAD surgery.

Several studies have reported higher early mortality associated with root replacement in an acute setting,^22^ whereas others have demonstrated comparable outcomes when performed selectively.^23^^,^^24^ These conflicting data underscore the need for a more refined, anatomy-driven approach rather than relying solely on diameter alone. In some Western centers, root intervention may be considered at diameters exceeding 45 mm, and valve-sparing techniques such as remodeling or reimplantation have been reported. However, their role in emergency ATAAD surgery remains uncertain. Because the dissected aortic wall is often fragile, operative simplicity and minimization of ischemic time are critical priorities. Therefore, routine root intervention at 45-49 mm might not be justified without significant structural destruction or valve pathology.

Collectively, these findings clarify the gray zone in aortic root management during ATAAD repair. A root diameter of 50 mm appears to represent a practical threshold beyond which root preservation becomes unreliable. Roots <50 mm, including those measuring 45 to 49 mm, can be safely preserved in selected patients. Patient age, comorbidities, and life expectancy also should be considered when determining the extent of root intervention.

In elderly patients and patients with limited life expectancy, a more conservative strategy may be appropriate, whereas a more aggressive approach may be justified in younger individuals with longer anticipated survival. Notably, younger patients in our cohort tended to present with larger initial root diameters. Although patients with known syndromic aortic disease were excluded, unrecognized genetic predisposition may have contributed to this observation. Routine genetic testing was not performed systematically in this emergency setting. Future studies incorporating structured genetic evaluation may further refine risk stratification in younger patients.

### Limitations

This study has several limitations. First, it was a retrospective single-center study, and the surgical strategy reflects the philosophy of a high-volume aortic center, which may limit the generalizability of our findings. Second, aortic root measurements may vary depending on the cardiac phase and hemodynamic conditions at imaging. Third, the number of patients with root diameters ≥50 mm was relatively small, and some patients with partial sinus destruction were included in the 45 to 49 mm group, introducing potential heterogeneity. Patients with connective tissue disorders were excluded; therefore, the proposed 50-mm threshold cannot be extrapolated to these populations.

## Conclusions

Aortic root diameter is an important determinant of long-term outcomes after ATAAD repair. Root preservation is safe and durable for root diameters <50 mm, even when the root diameter is 45 to 49 mm. However, once the root diameter reaches 50 mm or greater, the risk of progressive enlargement and need for reoperation increases sharply, supporting concomitant root replacement in these patients. These findings provide a practical criterion for intraoperative decision making and may contribute to a more precise surgical threshold for aortic root management in ATAAD.

## Conflict of Interest Statement

The authors reported no conflicts of interest.

The *Journal* policy requires editors and reviewers to disclose conflicts of interest and to decline handling or reviewing manuscripts for which they may have a conflict of interest. The editors and reviewers of this article have no conflicts of interest.
